# Supplementary material for: Trajectories of school absences across compulsory schooling and their impact on children’s academic achievement: An analysis based on linked longitudinal survey and school administrative data
Source: PLoS One. 2024 Aug 12;19(8):e0306716. doi: 10.1371/journal.pone.0306716 (PMC11318909; doi:10.1371/journal.pone.0306716)
Supplement: S1 File — (DOCX) [file pone.0306716.s001.docx]

## S1. Sample selection

**S1 Table**

*Diagram of sample restrictions*

| Millenium Cohort Study (N=19,244) |  |  |
| --- | --- | --- |
|  |  | **Excluded** (N=10,197)  Dropped out of MCS before sweep 3, or  residing in Northern Ireland, Scotland, or Wales |
| Residing in England at MCS sweep 3, 4, or 5 (N=9,047) |  |  |
|  |  | **Excluded** (N=558)  Did not consent to link data to NPD |
| Consented to link data to NPD (N=8,489) |  |  |
|  |  | **Excluded** (N=51)  Not linked to MCS |
| Linked with NPD data (N=8,438) |  |  |
|  |  | **Excluded** (N=232)  Linked in sweep 3 or 5 |
| Linked in sweep 4 (N=8,206) |  |  |
|  |  | **Excluded** (N=988)  Missing information on absences in a full academic year or GCSE achievement |
| Analysis sample (N=7,218) |  |  |
