# Supplementary material for: Trajectories of school absences across compulsory schooling and their impact on children’s academic achievement: An analysis based on linked longitudinal survey and school administrative data
Source: PLoS One. 2024 Aug 12;19(8):e0306716. doi: 10.1371/journal.pone.0306716 (PMC11318909; doi:10.1371/journal.pone.0306716)
Supplement: S2 File — (DOCX) [file pone.0306716.s002.docx]

## S2. Inverse probability weights

**S2 Table**

*Logit coefficients of regressing analysis sample participation on baseline covariates.*

|  | Coefficient | SE |
| --- | --- | --- |
|  |  |  |
| Date of Birth | 0.007 | (0.008) |
| Boy | -0.057 | (0.057) |
| **Ethnicity** |  |  |
| Mixed | -0.402*** | (0.137) |
| Indian | -0.373** | (0.153) |
| Pakistani/Bangladeshi | -0.736*** | (0.126) |
| Black | -0.666*** | (0.134) |
| Other | -0.185 | (0.209) |
| **Family structure** |  |  |
| Stepfamily | -0.036 | (0.149) |
| Single parent | -0.239** | (0.104) |
| Other | -0.662* | (0.402) |
| Household size | -0.070 | (0.047) |
| Number of children | 0.070 | (0.054) |
| **Parental education** |  |  |
| NVQ 1 | 0.156 | (0.151) |
| NVQ 2 | 0.252** | (0.109) |
| NVQ 3 | 0.006 | (0.122) |
| NVQ 4 | -0.185 | (0.113) |
| NVQ 5 | -0.577*** | (0.142) |
| **Parents’ social class** |  |  |
| NSSEC 2 | 0.396*** | (0.085) |
| NSSEC 3 | 0.608*** | (0.121) |
| NSSEC 4 | 0.210* | (0.127) |
| NSSEC 5 | 0.533*** | (0.148) |
| NSSEC 6 | 0.301** | (0.124) |
| NSSEC 7 | 0.441*** | (0.145) |
| Neighborhood deprivation | -0.016 | (0.013) |
| **Housing tenure** |  |  |
| Owned with mortgage | 0.487*** | (0.119) |
| Rent - Local authority | 0.105 | (0.149) |
| Rent - Housing association or private | -0.046 | (0.140) |
| Other | 0.201 | (0.213) |
| Income | -0.002*** | (0.000) |
| **Region** |  |  |
| Northwest | -0.227 | (0.178) |
| Yorkshire and the Humber | -0.092 | (0.183) |
| East Midlands | -0.210 | (0.189) |
| West Midlands | -0.332* | (0.178) |
| East of England | -0.202 | (0.181) |
| London | -0.474*** | (0.176) |
| Southeast | -0.412** | (0.174) |
| Southwest | -0.408** | (0.184) |
| Residential Moves | -0.260*** | (0.083) |
| Birthweight | 0.003 | (0.054) |
| **Birth complications** |  |  |
| Complications, not in special care | -0.045 | (0.063) |
| Complications, in special care | -0.098 | (0.108) |
| Alcohol during pregnancy | 0.045 | (0.027) |
| Smoking during pregnancy | -0.135 | (0.088) |
| Parental depression | 0.004 | (0.010) |
| General health | 0.000 | (0.037) |
| **Longstanding illness** |  |  |
| Yes, but at most a little bit affected | 0.008 | (0.088) |
| Yes, strongly affected | -0.565*** | (0.118) |
| Bracken (Age 3) | 0.004 | (0.002) |
| BAS vocabulary (Age 3) | 0.005 | (0.004) |
| Externalizing (Age 3) | 0.004 | (0.009) |
| Internalizing (Age 3) | -0.003 | (0.013) |
| Value of child independence | 0.003 | (0.029) |
| Value of child obedience | -0.036 | (0.030) |
| Constant | -1.792 | (3.970) |

*Note*. N=8,986, weighted with MCS weights. *** p<0.01, ** p<0.05, * p<0.1. Reference categories: Ethnicity: White, Family Structure: two natural parents, Parental Education: None, Parental social class: NSSEC 1, Housing tenure: Owned outright, Region: Northeast, Birth complications: No, Long-standing illness: No.
