# Supplementary material for: Trajectories of school absences across compulsory schooling and their impact on children’s academic achievement: An analysis based on linked longitudinal survey and school administrative data
Source: PLoS One. 2024 Aug 12;19(8):e0306716. doi: 10.1371/journal.pone.0306716 (PMC11318909; doi:10.1371/journal.pone.0306716)
Supplement: S3 File — (DOCX) [file pone.0306716.s003.docx]

## S3. Distribution of absences

**S3 Table**

*Distribution of absences by year and their correlations.*

| Year | Total absences | | | Authorized absences | | | Unauthorized absences | | | Correlations | | | |
| --- | --- | --- | --- | --- | --- | --- | --- | --- | --- | --- | --- | --- | --- |
|  | Mean | SD | Median | Mean | SD | Median | Mean | SD | Median | Total | Auth. | Unauth. | Auth.-Unauth. |
| 1 | .054 | .052 | .039 | .049 | .046 | .038 | .005 | .020 | .000 |  |  |  | .154 |
| 2 | .050 | .049 | .038 | .045 | .043 | .033 | .005 | .017 | .000 | .547 | .489 | .413 | .191 |
| 3 | .047 | .048 | .035 | .042 | .039 | .032 | .005 | .022 | .000 | .506 | .470 | .394 | .149 |
| 4 | .047 | .046 | .036 | .042 | .041 | .032 | .005 | .018 | .000 | .540 | .499 | .420 | .131 |
| 5 | .045 | .045 | .033 | .039 | .038 | .029 | .006 | .022 | .000 | .558 | .530 | .436 | .085 |
| 6 | .039 | .042 | .026 | .034 | .037 | .023 | .005 | .016 | .000 | .561 | .550 | .350 | .122 |
| 7 | .045 | .052 | .032 | .040 | .042 | .029 | .006 | .022 | .000 | .568 | .521 | .314 | .200 |
| 8 | .045 | .057 | .029 | .037 | .043 | .026 | .008 | .032 | .000 | .618 | .559 | .551 | .136 |
| 9 | .053 | .068 | .034 | .042 | .047 | .029 | .011 | .043 | .000 | .635 | .579 | .555 | .159 |
| 10 | .056 | .082 | .032 | .041 | .054 | .026 | .015 | .052 | .000 | .622 | .545 | .474 | .198 |
| 11 | .065 | .103 | .036 | .045 | .062 | .029 | .020 | .071 | .000 | .658 | .525 | .616 | .177 |

*Note*. N=7,218. Weighted. The row “Correlations: Total” show the correlations between total absences in one year and total absences in the previous year. The rows “Correlation: Auth.” And “Correlation: Unauth.” Show the same for authorized and unauthorized absences. The row “Correlations: Auth.-Unauth” shows the correlation between authorized and unauthorized absences in the same year.
