# Supplementary material for: Trajectories of school absences across compulsory schooling and their impact on children’s academic achievement: An analysis based on linked longitudinal survey and school administrative data
Source: PLoS One. 2024 Aug 12;19(8):e0306716. doi: 10.1371/journal.pone.0306716 (PMC11318909; doi:10.1371/journal.pone.0306716)
Supplement: S4 File — (DOCX) [file pone.0306716.s004.docx]

## S4. Timing of measurement of covariates

**S4 Table**

*Measurement timing of covariates*

| **Dimension** | **Variable** | **Age 1**  **Sweep 1** | **Age 3**  **Sweep 2** | **Age 5**  **Sweep 3** | **Age 7**  **Sweep 4** | **Age 11**  **Sweep 5** | **Age 14**  **Sweep 6** |
| --- | --- | --- | --- | --- | --- | --- | --- |
| **SES** | Highest parental education – NVQ |  |  | **B** |  |  |  |
|  | Highest NSSEC 7 |  |  | **B** |  |  |  |
|  | Household income |  |  | **B** |  |  |  |
|  | Housing tenure |  |  | **B** |  |  |  |
|  | Neighbourhood deprivation decile |  |  | **B** |  |  |  |
| **Demographics** | Ethnicity | **B** |  |  |  |  |  |
|  | Date of birth | **B** |  |  |  |  |  |
|  | Gender | **B** |  |  |  |  |  |
|  | Family structure |  |  | **B** |  |  |  |
|  | Household size |  |  | **B** |  |  |  |
|  | Region within England |  |  |  | **B** |  |  |
|  | Number of children in household |  |  | **B** |  |  |  |
| **Cognitive Abilities / Achievement** | BAS vocabulary + Bracken |  | **B** |  |  |  |  |
|  | BAS vocabulary + BAS pattern + BAS picture |  |  | **R** |  |  |  |
|  | Math + BAS reading + BAS pattern |  |  |  | **R** |  |  |
|  | BAS Verbal similarities |  |  |  |  | **R** |  |
|  | Vocabulary |  |  |  |  |  | **R** |
|  | Key stage English |  |  |  | **R** | **R** |  |
|  | Key stage Math |  |  |  | **R** | **R** |  |
| **Attitude towards school** | CMs attitude towards school |  |  |  | **R** | **R** | **R** |
|  | CMs attitude towards school (reported by parents) |  |  | **R** |  |  |  |
|  | CMs educational aspirations |  |  |  |  |  | **R** |
|  | Parents educational aspirations |  |  |  | **R** | **R** |  |
| **Child behaviour** | Internalizing |  | **B** | **R** | **R** | **R** | **R** |
|  | Externalizing |  | **B** | **R** | **R** | **R** | **R** |
| **Child Health** | Child has longstanding illness |  |  | **B** |  |  |  |
|  | General health |  |  | **B** |  |  |  |
| **Birth conditions** | Birthweight | **B** |  |  |  |  |  |
|  | Was in special care unit | **B** |  |  |  |  |  |
|  | Mother smoked during pregnancy | **B** |  |  |  |  |  |
|  | Mother’s alcohol consumption during pregnancy | **B** |  |  |  |  |  |
| **Parental involvement** | Parents had meeting with teacher |  |  | **R** | **R** | **R** | **R** |
|  | Joint learning-related activities – sum score |  |  | **R** | **R** |  |  |
| **School characteristics** | Stream |  |  |  | **R** | **R** |  |
|  | Set (English and Math) |  |  |  | **R** | **R** |  |
|  | School fees |  |  | **R** | **R** | **R** | **R** |
| **Disruptive events** | Parents mental health problems |  |  | **B** |  |  |  |
|  | Changed school |  |  | **R** | **R** | **R** | **R** |
|  | Moved residence |  |  | **B** |  |  |  |

*Note*. B indicates that the variable is included as a baseline confounder, R indicates that the variable is included as a residualized confounder.
