# Supplementary material for: Trajectories of school absences across compulsory schooling and their impact on children’s academic achievement: An analysis based on linked longitudinal survey and school administrative data
Source: PLoS One. 2024 Aug 12;19(8):e0306716. doi: 10.1371/journal.pone.0306716 (PMC11318909; doi:10.1371/journal.pone.0306716)
Supplement: S5 File — (DOCX) [file pone.0306716.s005.docx]

## S5. Measurement of latent factors

**S5 Table**

*Measurement of latent factors not provided by the MCS*.

| Construct | Items | Cronbach’s alpha | Method | Resulting scale |
| --- | --- | --- | --- | --- |
| Attitude towards school  reported by parents (Sweep 3) | Whether CM enjoys school  How often CM talks about school  How often reluctant to go to school | 0.49 | Sum score | 1 (highest motivation) to  6 (lowest motivation) |
| Attitude towards school  reported by cohort member (Sweep 4) | How often do you try to do your best at school?  How often is school interesting?  How often do you feel unhappy at school?  How often do you get tired at school?  How often do you get fed up at school? | 0.56 | Sum score | 1 (lowest motivation) to  3 (highest motivation) |
| Attitude towards school  reported by cohort member (Sweep 5) | How often do you try your best at school?  How often do you find school interesting?  How often do you feel unhappy at school?  How often do you get tired at school?  How often do you feel school is a waste of time? | 0.71 | Sum score | 1 (lowest motivation) to  4 (highest motivation) |
| Attitude towards school  reported by cohort member (Sweep 6) | How often do you try your best at school?  How often do you find school interesting?  How often do you feel unhappy at school?  How often do you get tired at school?  How often do you feel school is a waste of time?  How often difficult to keep mind on work at school? | 0.75 | Sum score | 1 (lowest motivation) to  4 (highest motivation) |
| Joint parent-child activities (Sweep 3) | How often do you read to CM?  How often tells stories to CM?  How often does musical activities with CM?  How often does CM paint/draw at home? | 0.58 | Sum score | 1 (fewest activities) to  6 (most activities) |
| Joint parent-child activities (Sweep 4) | How often do you read to CM?  How often tells stories to CM?  How often does musical activities with CM?  How often does CM paint/draw at home? | 0.57 | Sum score | 1 (fewest activities) to  6 (most activities) |

*Note*. CM=Cohort Member.
