# Supplementary material for: Trajectories of school absences across compulsory schooling and their impact on children’s academic achievement: An analysis based on linked longitudinal survey and school administrative data
Source: PLoS One. 2024 Aug 12;19(8):e0306716. doi: 10.1371/journal.pone.0306716 (PMC11318909; doi:10.1371/journal.pone.0306716)
Supplement: S6 File — (DOCX) [file pone.0306716.s006.docx]

## S6. Distribution of achievement and covariates

**S6 Table**

*Distribution of achievement and covariates*.

|  | Proportion/Mean | SD |
| --- | --- | --- |
| **Achievement** |  |  |
| 5 or more GCSEs | .588 | - |
| Attainment 8 | 47.308 | 19.117 |
| English | 10.082 | 4.151 |
| Math | 9.203 | 4.320 |
| **Baseline Confounders** |  |  |
| *Ethnicity* |  |  |
| White | .828 | - |
| Mixed | .039 | - |
| Indian | .023 | - |
| Pakistani/Bangladeshi | .055 | - |
| Black | .039 | - |
| Other | .016 | - |
| *Family Structure* |  |  |
| Two natural parents | .737 | - |
| Stepfamily | .057 | - |
| Single parent | .201 | - |
| Other | .004 | - |
| *Parental Education^1^* |  |  |
| None | .122 | - |
| NVQ 1 | .065 | - |
| NVQ 2 | .256 | - |
| NVQ 3 | .149 | - |
| NVQ 4 | .341 | - |
| NVQ 5 | .066 | - |
| *Parental class^2^* |  |  |
| NSSEC 1 | .157 | - |
| NSSEC 2 | .286 | - |
| NSSEC 3 | .142 | - |
| NSSEC 4 | .085 | - |
| NSSEC 5 | .082 | - |
| NSSEC 6 | .151 | - |
| NSSEC 7 | .097 | - |
| *Housing tenure* |  |  |
| Owned outright | .048 | - |
| Owned with mortgage | .580 | - |
| Rent - Local authority | .155 | - |
| Rent - Housing association or private | .190 | - |
| Other | .027 | - |
| *Region* |  |  |
| Northeast | .045 | - |
| Northwest | .132 | - |
| Yorkshire and the Humber | .107 | - |
| East Midlands | .088 | - |
| West Midlands | .100 | - |
| East of England | .119 | - |
| London | .134 | - |
| Southeast | .175 | - |
| Southwest | .101 | - |
| *Birth complications* |  |  |
| No complications | .536 | - |
| Complications, not in special care | .377 | - |
| Complications, in special care | .087 | - |
| *Long-standing illness* |  |  |
| No | .802 | - |
| Yes, but at most a little bit affected | .139 | - |
| Yes, strongly affected | .059 | - |
| Date of birth^3^ | 493.448 | 3.466 |
| Boy | .512 | - |
| HH size | 4.296 | 1.240 |
| Number of children in HH | 2.391 | 1.045 |
| Neighborhood deprivation decile^4^ | 5.338 | 2.957 |
| HH income^5^ | 356.859 | 220.246 |
| Residential Moves^6^ | .134 | - |
| Birthweight (in kg) | 3.333 | .590 |
| Alcohol during pregnancy^7^ | 6.380 | 1.073 |
| Smoking during pregnancy^8^ | .223 | - |
| Parental depression^9^ | 3.386 | 3.359 |
| Child general health^10^ | 1.730 | .854 |
| Bracken (Age 3)^11^ | 103.355 | 16.162 |
| BAS vocabulary (Age 3)^12^ | 48.735 | 11.326 |
| Externalizing (Age 3)^13^ | 6.940 | 3.845 |
| Internalizing (Age 3)^14^ | 3.017 | 2.546 |
| Parents value child independence^15^ | 2.059 | 1.228 |
| Parents value child obedience^15^ | 3.220 | 1.134 |
| **Time-varying confounders** | |  |
| BAS picture (Age 5)^16^ | 54.986 | 10.377 |
| BAS vocabulary (Age 5) | 53.960 | 11.473 |
| BAS pattern (Age 5)^17^ | 50.414 | 10.077 |
| Externalizing (Age 5) | 4.842 | 3.353 |
| Internalizing (Age 5) | 2.583 | 2.543 |
| Educational motivation (Age 5, reported by parents) | 1.583 | .714 |
| Parents met teacher (Age 5)^18^ | .920 | - |
| Joint parent-child activities (Age 5) | 4.297 | .848 |
| School Fees (Age 5) | .995 | - |
| School change (Age 5) | .031 | - |
| BAS reading (Age 7)^19^ | 112.306 | 17.447 |
| BAS pattern (Age 7) | 52.670 | 10.817 |
| NFER math (Age 7)^20^ | 97.618 | 15.508 |
| Externalizing (Age 5) | 4.808 | 3.532 |
| Internalizing (Age 5) | 2.838 | 2.824 |
| Educational motivation (Age 7) | 2.350 | .383 |
| Parents met teacher (Age 7) | .951 | - |
| Reading score in KS 1^21^ | 15.990 | 4.118 |
| Writing score in KS 1 | 14.574 | 3.873 |
| Math score in KS 1 | 16.168 | 3.577 |
| Parents' educational aspiration (Age 7)^22^ | .979 | - |
| Joint parent-child activities (Age 7) | 3.921 | .908 |
| School Fees (Age 7) | *N<10* | - |
| School change (Age 7) | .107 | - |
| Top Stream (Age 7)^23^ | .073 | - |
| Top English set (Age 7)^24^ | .116 | - |
| Top Math set (Age 7) | .152 | - |
| Bottom Stream (Age 7) | .043 | - |
| Bottom English set (Age 7) | .072 | - |
| Bottom Math set (Age 7) | .085 | - |
| BAS Verbal similarities (Age 11)^25^ | 58.476 | 9.492 |
| Externalizing (Age 11) | 6.509 | 1.933 |
| Internalizing (Age 11) | 6.656 | 2.612 |
| Educational motivation (Age 11) | 3.186 | .477 |
| Parents met teacher (Age 11) | .955 | - |
| Reading score in KS 2^26^ | 28.724 | 4.258 |
| Math score in KS 2 | 28.918 | 4.720 |
| Parents' educational aspiration (Age 11) | .883 | - |
| No School Fees (Age 11) | .998 | - |
| School change (Age 11) | .211 | - |
| Top Stream (Age 11) | .099 | - |
| Top English set (Age 11) | .199 | - |
| Top Math set (Age 11) | .310 | - |
| Bottom Stream (Age 11) | .038 | - |
| Bottom English set (Age 11) | .091 | - |
| Bottom Math set (Age 11) | .143 | - |
| Vocabulary (Age 14)^27^ | 6.986 | 2.579 |
| Externalizing (Age 14) | 4.473 | 3.583 |
| Internalizing (Age 14) | 3.763 | 3.355 |
| Educational motivation (Age 14) | 2.896 | .491 |
| Parents met teacher (Age 14) | .900 | - |
| Child's educational aspiration (Age 14)^28^ | 86.431 | 20.494 |
| No School Fees (Age 14) | *N<10* | - |
| School change (Age 14) | .065 | - |

Note. N=7,218. Multiple imputed and weighted. Proportions for “School fees (Age 7)” and “School fees (Age 14)” have been suppressed to comply with the requirements for the statistical disclosure review check of the UK Data Service. Standard deviations are not reported for categorical variables. GCSE Attainment in English and math ranges from 0 to 18 because they are counted double for Attainment 8.

**1 Parental education:** National Vocational Qualification (NVQ)

NVQ 5: Higher Degree and Postgraduate qualifications or vocational equivalent

NVQ 4: First Degree, diploma in higher education or vocational equivalent

NVQ 3: A/AS/S Levels/SCE Higher or vocational equivalent

NVQ 2: O Level or GCSE grade A-C or vocational equivalent

NVQ 1: CSE below grade 1/GCSE or O Level below grade C or vocational equivalent

**2 Social class:** National Statistics Socio-economic classification (NS-SEC)

NS-SEC 1: Higher managerial, administrative and professional occupations

NS-SEC 2: Lower managerial, administrative and professional occupations

NS-SEC 3: Intermediate occupations

NS-SEC 4: Small employers and own account workers

NS-SEC 5: Lower supervisory and technical occupations

NS-SEC 6: Semi-routine occupations

NS-SEC 7: Routine occupations

**3 Date of birth:** In months since January 1960.

**4 Neighborhood deprivation**: Decile of the index of multiple deprivation at Lower Super Output Area Level.

**5 HH income**: OECD equivalized income. Provided by MCS.

**6 Residential moves:** Whether the child moved residence in 2005 or 2006 (1) or not (0).

**7 Alcohol during pregnancy:** Frequency of alcohol consumption during pregnancy (1 “Every day”, 2 “5-6 times per week”, 3 “3-4 times per week”, 4 “1-2 times per week”, 5 “1-2 times per months”, 6 “Less than once a month”, 7 “Never”).

**8 Smoking during pregnancy:** Whether mother smoked during pregnancy (1) or not (0).

**9 Parental depression:** Score on Kessler scale, range 0-24, provided by MCS, averaged over parents.

**10 Child general health:** Child’s general level of health as reported by parents (1 “excellent” 2 “very good” 3 “good” 4 “fair or poor”)

**11 Bracken (Age 3):** Measures basic knowledge in preparation for school. The test includes 88 items, belonging to 6 different sub-set (colours, letters, numbers/counting,sizes, comparisons, shapes). Ability adjusted scores. Range: 56-149. Provided by MCS (see Hansen, 2017).

**12 BAS Vocabulary (Age 3, Age 5):** Measures expressive verbal ability. Children are shown up to 36 pictures of objects and are asked to name them. Ability and age adjusted. Range 20-80. Provided by MCS (see Hansen, 2017).

**13 Externalizing (Age 3, 5, 7, 11, 14):** Raw score of the conduct problems subscale plus the hyperactivity subscale of the strengths and difficulties questionnaire (Goodman, 1997). Range 0-20.

**14 Internalizing (Age 3, 5, 7, 11, 14):** Raw score of the emotion symptoms subscale plus the peer problems subscale of the strengths and difficulties questionnaire (Goodman, 1997). Range 0-20.

**15 Parents value child independence/obedience:** Parents were asked about the most, second most, third most, and least important value to install in their child. They could choose from: 1 “To be well liked or popular”, 2 “To think for himself or herself” (child independence), 3 “To work hard”, 4 “To help others when they need help”, 5 “To obey his or her parents” (obedience), 6 “To learn religious values”. If independence or obedience were mentioned as most important, they were coded as 1, second-most important as 2, third most-important as 3, and least important as 5. When independence and obedience were not mentioned as any of the aforementioned options, they were coded as 4.

**16 BAS picture (Age 5):** Measures children’s problem-solving skills. Children are shown a row of four pictures and are asked to choose a fifth picture that matches the other pictures best. Ability and age adjusted. Range 20-80. Provided by MCS (see Hansen, 2017).

**17 BAS pattern (Age 5, 7):** Children were presented different patterns and are asked to replicate the pattern. 23 Items. Ability and age adjusted. Range 20-80. Provided by MCS (see Hansen, 2017).

**18 Parents met teacher (Age 5):** Whether parents have attended the parent evening in school.

**19 BAS reading (Age 7):** Measures English reading ability. Children are asked to read words to the interviewer. Ability and age adjusted. Range 55-145. Provided by MCS (see Hansen, 2017).

**20 NFER math (Age 7):** Measures mathematical skills and knowledge. Covers numbers, shapes and data handling. Ability and age adjusted. Range 69-136. Provided by MCS (see Hansen, 2017).

**21 Reading/Writing/Math score in KS 1:** Teacher assigned reading score at the end of year 2. Range 3-39.

**22 Parents' educational aspiration (Age 7, 11):** Whether parents want to child to continue full-time education at age 16 (i.e., after mandatory school attendance).

**23 Bottom/Top Stream (Age 7, 11):** Whether the child attends the bottom or top stream if the child is streamed. Reported by teachers.

**24 Bottom/Top English/Math set (Age 7, 11):** Whether the child is in the bottom or top set in English and Math if there are sets in English and Math. Reported by teachers.

**25 BAS Verbal similarities (Age 11):** Assess verbal reasoning and verbal knowledge. The interviewer reads out three words to the child and the child is asked to say what these words have in common. Ability and age adjusted. Range 20-80. Provided by MCS (see Hansen, 2017).

**26 Reading/Math score in KS 2:** Fine graded score of standardized tests at the end of year 6. Range 3-39.

**27 Vocabulary (Age 14):** Children were presented words and are then offered a multiple-choice list of 5 words from which they should pick the word with the same meaning. Raw scores. Range 0-19. Provided by MCS.

**28 Child’s educational aspiration (Age 14):** How likely the child thinks that they will remain in school at the end of year 11 on a scale from 0 to 100.

# **References**

Goodman, R. (1997). The Strengths and Difficulties Questionnaire: A Research Note. *Journal of Child Psychology and Psychiatry*, 38, 581-586.

Hansen, K. (2014). Millennium Cohort Study. A Guide to the Datasets. First, second, third, fourth and fifth surveys. Centre for Longitudinal Studies. Institute of Education, University of London.
