# Supplementary material for: Trajectories of school absences across compulsory schooling and their impact on children’s academic achievement: An analysis based on linked longitudinal survey and school administrative data
Source: PLoS One. 2024 Aug 12;19(8):e0306716. doi: 10.1371/journal.pone.0306716 (PMC11318909; doi:10.1371/journal.pone.0306716)
Supplement: S7 File — (DOCX) [file pone.0306716.s007.docx]

## S7. Number of clusters

Existing research cannot deduce the optimal number of clusters. Therefore, we implemented k-means for longitudinal data (KML) with two to eight clusters (henceforth k) and compared solutions in terms of fit indices, predictive validity, number of cases in the smallest cluster, and interpretability.

The kml3d-package provides various fit indices to determine the optimal number of clusters: the Calinski-Harabasz index, the Ray-Turi index, the Davies-Bouldin index, AIC and BIC (see Genolini et al., 2015 for more information on fit indices). Higher values for all indices indicate a better fit. Table F1 displays the various fit indices for solutions with two to eight clusters.

As in previous applied studies with larger sample sizes, fit indices are inconsistent and do not point to a single preferred k (Ciarrochi et al. 2019; Chow et al. 2022). Ray-Turi and Davies-Bouldin propose k = 2, Calinski and Harabasz propose k = 5, while AIC and BIC propose k = 8 as preferable solutions.

Figure F1 depicts the proportion of variance in achievement measures that can be explained by absence trajectories in bivariate linear regressions. When using k=7 or k=8, the most variance in achievement can be explained. 40-60% less variance is explained when k = 2, 15-20% less when k = 3, and 3-10% less when k = 4, k = 5, or k = 6.

Tables F2 and F3 display the number and proportion of students in each cluster, respectively. With k = 2, one cluster contains 96.81% of the students in the weighted sample, while the other cluster contains 3.12% of the students. When more than five clusters are utilized, the smallest cluster contains fewer than 0.5% of pupils.

Overall, k=5 is our preferred cluster solution. It ranks first on the Calinksi-Harabasz index and second on the Davies-Boulding index. Using k=5 significantly increases the variance in achievement that is explained in comparison to k=2 or k=3 and explains nearly as much variance as k=7 or k=8. With k equal to five, the smallest cluster is nearly twice as large as it would be with six or more clusters.

**S7 Table 1**

*Fit indices of cluster solutions with different numbers of clusters (k)*

| k | Calinski-Harabasz | Ray-Turi | Davies-Bouldin | AIC | BIC |
| --- | --- | --- | --- | --- | --- |
| 2 | 1834.218 | -0.00533 | -1.27728 | -414656 | -414966 |
| 3 | 2295.827 | -0.04682 | -1.48302 | -398021 | -398483 |
| 4 | 2288.85 | -0.04761 | -1.52709 | -389843 | -390456 |
| 5 | 2320.198 | -0.06145 | -1.3505 | -381109 | -381873 |
| 6 | 2287.427 | -0.05989 | -1.51992 | -374784 | -375700 |
| 7 | 2301.381 | -0.05881 | -1.40028 | -370406 | -371473 |
| 8 | 2303.352 | -0.05754 | -1.4133 | -369108 | -370327 |

*Note***.** N=7,218, unweighted.

**S7 Figure**

*Explained variance in bivariate regressions with different numbers of clusters (k).*


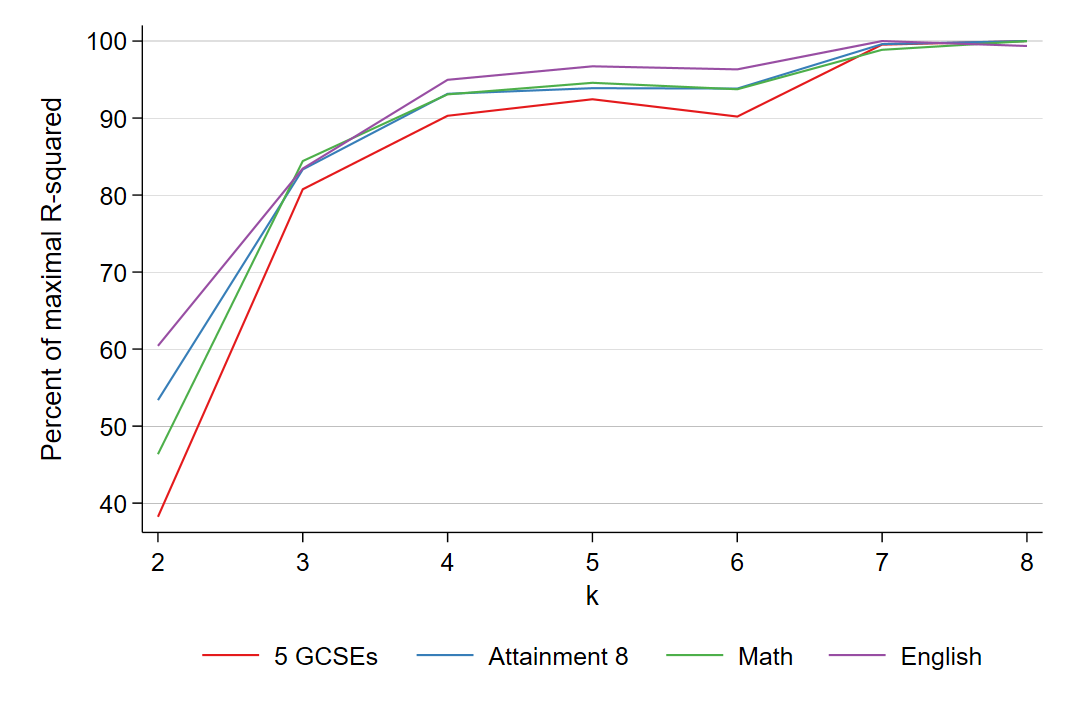


*Note.* Values are rescaled relative to the maximal R-squared separately by outcome. N=7,218, weighted.

**S7 Table 2**

*Percent of observations in clusters with different numbers of clusters (k).*

|  | k=2 | k=3 | k=4 | k=5 | k=6 | k=7 | k=8 |
| --- | --- | --- | --- | --- | --- | --- | --- |
| Cluster 1 | 96.81 | 72.94 | 66.52 | 66.33 | 66.62 | 55.17 | 48.80 |
| Cluster 2 | 3.19 | 25.04 | 28.33 | 27.70 | 27.96 | 26.03 | 18.61 |
| Cluster 3 |  | 2.02 | 4.23 | 3.53 | 2.78 | 13.69 | 19.47 |
| Cluster 4 |  |  | 0.91 | 1.62 | 1.62 | 2.29 | 8.61 |
| Cluster 5 |  |  |  | 0.82 | 0.58 | 1.41 | 2.08 |
| Cluster 6 |  |  |  |  | 0.45 | 1.04 | 1.04 |
| Cluster 7 |  |  |  |  |  | 0.36 | 1.04 |
| Cluster 8 |  |  |  |  |  |  | 0.36 |

*Note*. N=7,218, weighted.

**S7 Table 3**

*Number of observations in clusters with different numbers of clusters (k).*

|  | k=2 | k=3 | k=4 | k=5 | k=6 | k=7 | k=8 |
| --- | --- | --- | --- | --- | --- | --- | --- |
| Cluster 1 | 6992 | 5338 | 4841 | 4825 | 4848 | 4039 | 3526 |
| Cluster 2 | 226 | 1736 | 2025 | 1984 | 2001 | 1836 | 1431 |
| Cluster 3 |  | 144 | 282 | 241 | 190 | 990 | 1360 |
| Cluster 4 |  |  | 70 | 108 | 109 | 152 | 588 |
| Cluster 5 |  |  |  | 60 | 43 | 95 | 137 |
| Cluster 6 |  |  |  |  | 27 | 79 | 79 |
| Cluster 7 |  |  |  |  |  | 27 | 70 |
| Cluster 8 |  |  |  |  |  |  | 27 |

*Note*. N=7,218, unweighted.

# **References**

Chow, A. R., Pingault, J. B., & Baldwin, J. R. (2022). Early risk factors for joint trajectories of bullying victimisation and perpetration. *European Child & Adolescent Psychiatry*, 1-9.

Ciarrochi, J., Sahdra, B. K., Hawley, P. H., & Devine, E. K. (2019). The upsides and downsides of the dark side: A longitudinal study into the role of prosocial and antisocial strategies in close friendship formation. *Frontiers in Psychology*, *10*, 114.
