# Supplementary material for: Trajectories of school absences across compulsory schooling and their impact on children’s academic achievement: An analysis based on linked longitudinal survey and school administrative data
Source: PLoS One. 2024 Aug 12;19(8):e0306716. doi: 10.1371/journal.pone.0306716 (PMC11318909; doi:10.1371/journal.pone.0306716)
Supplement: S8 File — (DOCX) [file pone.0306716.s008.docx]

## S8. Yearly mean absences in preferred cluster solution

**S8 Table**

*Mean authorized and unauthorized absences by absences trajectory cluster (proportions).*

|  | Authorized | | | | | Unauthorized | | | | |
| --- | --- | --- | --- | --- | --- | --- | --- | --- | --- | --- |
| Year | CLA | CMAA | MIUA | SIAA | SIUA | CLA | CMAA | MIUA | SIAA | SIUA |
| 1 | .034 | .080 | .083 | .065 | .073 | .002 | .007 | .025 | .004 | .024 |
| 2 | .030 | .076 | .076 | .064 | .069 | .002 | .008 | .023 | .006 | .019 |
| 3 | .027 | .070 | .071 | .064 | .067 | .003 | .008 | .029 | .007 | .019 |
| 4 | .027 | .070 | .077 | .064 | .074 | .003 | .009 | .024 | .010 | .018 |
| 5 | .025 | .065 | .073 | .079 | .071 | .003 | .009 | .027 | .008 | .024 |
| 6 | .020 | .058 | .069 | .077 | .077 | .003 | .007 | .023 | .011 | .033 |
| 7 | .024 | .066 | .085 | .114 | .078 | .002 | .008 | .038 | .027 | .056 |
| 8 | .022 | .061 | .078 | .135 | .083 | .003 | .010 | .059 | .020 | .132 |
| 9 | .025 | .067 | .081 | .164 | .099 | .003 | .011 | .102 | .039 | .173 |
| 10 | .024 | .061 | .071 | .258 | .133 | .004 | .015 | .141 | .043 | .297 |
| 11 | .029 | .060 | .076 | .344 | .086 | .006 | .017 | .167 | .054 | .623 |
| Average | .026 | .067 | .076 | .130 | .083 | .003 | .010 | .060 | .021 | .129 |

*Note*. N=7,218, weighted. CLA=Consistently Low Absence, CMAA=Consistently Moderate Authorized Absences, MIUA=Moderately Increasing Unauthorized Absences, SIAA=Strongly Increasing Authorized Absences, SIUA=Strongly Increasing Unauthorized Absences
