# Supplementary material for: Trajectories of school absences across compulsory schooling and their impact on children’s academic achievement: An analysis based on linked longitudinal survey and school administrative data
Source: PLoS One. 2024 Aug 12;19(8):e0306716. doi: 10.1371/journal.pone.0306716 (PMC11318909; doi:10.1371/journal.pone.0306716)
Supplement: S9 File — (DOCX) [file pone.0306716.s009.docx]

## S9. Bivariate associations between absence trajectories and achievement

**S9 Table 1**

*Average achievement by absence trajectory.*

|  | 5 GCSEs | | Attainment 8 | | English | | Math | |
| --- | --- | --- | --- | --- | --- | --- | --- | --- |
|  | Proportion | SD | Mean | SD | Mean | SD | Mean | SD |
| CLA | .682 | .466 | .229 | .919 | .186 | .933 | .218 | .948 |
| CMAA | .464 | .499 | -.269 | .915 | -.193 | .933 | -.284 | .914 |
| MIUA | .113 | .316 | -1.164 | .850 | -1.026 | .958 | -1.065 | .774 |
| SIAA | .230 | .421 | -1.176 | 1.103 | -1.050 | 1.208 | -.868 | 1.006 |
| SIUA | .000 | .000 | -2.075 | .609 | -2.025 | .693 | -1.755 | .641 |

*Note*. N=7,218, weighted. CLA=Consistently Low Absence, CMAA=Consistently Moderate Authorized Absences, MIUA=Moderately Increasing Unauthorized Absences, SIAA=Strongly Increasing Authorized Absences, SIUA=Strongly Increasing Unauthorized Absences. Mean values of Attainment 8, English, and Math scores are on the z-standardized scale.
