# Supplementary material for: Trajectories of school absences across compulsory schooling and their impact on children’s academic achievement: An analysis based on linked longitudinal survey and school administrative data
Source: PLoS One. 2024 Aug 12;19(8):e0306716. doi: 10.1371/journal.pone.0306716 (PMC11318909; doi:10.1371/journal.pone.0306716)
Supplement: S10 File — (DOCX) [file pone.0306716.s010.docx]

## S10. Full regression tables

**S10 Table**

*Full coefficient table of the regressions of achievement on absences trajectory cluster, baseline control variables, and residualized time-varying confounders*

|  | 5 GCSEs | | Attainment 8 | | English | | Math | |
| --- | --- | --- | --- | --- | --- | --- | --- | --- |
|  | b | SE | b | SE | b | SE | b | SE |
|  |  |  |  |  |  |  |  |  |
| *Absence trajectory clusters* |  |  |  |  |  |  |  |  |
| CMAA | -0.087*** | (0.012) | -0.206*** | (0.017) | -0.125*** | (0.020) | -0.212*** | (0.017) |
| MIUA | -0.271*** | (0.024) | -0.704*** | (0.052) | -0.618*** | (0.061) | -0.614*** | (0.045) |
| SIAA | -0.286*** | (0.040) | -1.004*** | (0.083) | -0.864*** | (0.091) | -0.721*** | (0.077) |
| SIUA | -0.331*** | (0.042) | -1.476*** | (0.097) | -1.432*** | (0.108) | -1.233*** | (0.110) |
| Date of Birth | -0.007*** | (0.001) | -0.015*** | (0.002) | -0.017*** | (0.002) | -0.011*** | (0.002) |
| Male child | -0.087*** | (0.009) | -0.216*** | (0.014) | -0.379*** | (0.017) | 0.025* | (0.014) |
| *Ethnicity* |  |  |  |  |  |  |  |  |
| Mixed | 0.030 | (0.025) | 0.078* | (0.044) | 0.104** | (0.047) | 0.037 | (0.044) |
| Indian | 0.165*** | (0.029) | 0.439*** | (0.053) | 0.361*** | (0.058) | 0.431*** | (0.055) |
| Pakistani/Bangladeshi | 0.168*** | (0.024) | 0.428*** | (0.034) | 0.518*** | (0.040) | 0.357*** | (0.035) |
| Black | 0.094*** | (0.028) | 0.195*** | (0.043) | 0.328*** | (0.051) | 0.092** | (0.042) |
| Other | 0.215*** | (0.042) | 0.494*** | (0.064) | 0.507*** | (0.077) | 0.465*** | (0.070) |
| *Family Structure* |  |  |  |  |  |  |  |  |
| Stepfamily | -0.062*** | (0.023) | -0.166*** | (0.036) | -0.118*** | (0.042) | -0.127*** | (0.037) |
| Single parent | -0.020 | (0.018) | -0.083*** | (0.027) | -0.059* | (0.031) | -0.058** | (0.027) |
| Other | -0.183** | (0.076) | -0.397*** | (0.136) | -0.378*** | (0.141) | -0.458*** | (0.134) |
| Household size | -0.000 | (0.010) | 0.002 | (0.014) | 0.008 | (0.016) | 0.011 | (0.014) |
| Number of children | -0.003 | (0.011) | -0.009 | (0.016) | -0.022 | (0.018) | -0.009 | (0.016) |
| *Parental Education* |  |  |  |  |  |  |  |  |
| NVQ 1 | -0.075*** | (0.024) | -0.112*** | (0.038) | -0.111** | (0.046) | -0.098*** | (0.037) |
| NVQ 2 | 0.009 | (0.018) | 0.025 | (0.029) | 0.027 | (0.034) | 0.039 | (0.027) |
| NVQ 3 | 0.039* | (0.021) | 0.088*** | (0.032) | 0.080** | (0.038) | 0.109*** | (0.031) |
| NVQ 4 | 0.080*** | (0.020) | 0.215*** | (0.031) | 0.188*** | (0.037) | 0.222*** | (0.030) |
| NVQ 5 | 0.119*** | (0.026) | 0.464*** | (0.042) | 0.371*** | (0.052) | 0.397*** | (0.046) |
| *Parental class* |  |  |  |  |  |  |  |  |
| NSSEC 2 | -0.021 | (0.015) | -0.157*** | (0.024) | -0.089*** | (0.029) | -0.202*** | (0.026) |
| NSSEC 3 | -0.047** | (0.020) | -0.242*** | (0.031) | -0.154*** | (0.036) | -0.304*** | (0.031) |
| NSSEC 4 | -0.050** | (0.024) | -0.253*** | (0.037) | -0.191*** | (0.042) | -0.289*** | (0.038) |
| NSSEC 5 | -0.068*** | (0.023) | -0.216*** | (0.034) | -0.158*** | (0.040) | -0.298*** | (0.036) |
| NSSEC 6 | -0.090*** | (0.021) | -0.257*** | (0.033) | -0.193*** | (0.040) | -0.316*** | (0.033) |
| NSSEC 7 | -0.065*** | (0.024) | -0.210*** | (0.037) | -0.152*** | (0.044) | -0.238*** | (0.037) |
| Neighborhood deprivation | 0.007*** | (0.002) | 0.010*** | (0.003) | 0.016*** | (0.004) | 0.012*** | (0.003) |
| *Housing tenure* |  |  |  |  |  |  |  |  |
| Owned with mortgage | -0.061*** | (0.019) | -0.106*** | (0.033) | -0.089** | (0.041) | -0.109*** | (0.035) |
| Rent - Local authority | -0.133*** | (0.025) | -0.222*** | (0.041) | -0.201*** | (0.050) | -0.178*** | (0.041) |
| Rent - Housing association or private | -0.133*** | (0.024) | -0.207*** | (0.039) | -0.186*** | (0.048) | -0.185*** | (0.039) |
| Other | -0.102*** | (0.039) | -0.204*** | (0.061) | -0.232*** | (0.075) | -0.144** | (0.059) |
| Household income | 0.000*** | (0.000) | 0.000*** | (0.000) | 0.000*** | (0.000) | 0.000*** | (0.000) |
| *Region* |  |  |  |  |  |  |  |  |
| North West | 0.006 | (0.023) | -0.074** | (0.036) | 0.000 | (0.044) | -0.062* | (0.036) |
| Yorkshire and the Humber | -0.017 | (0.024) | -0.044 | (0.036) | -0.055 | (0.045) | -0.017 | (0.037) |
| East Midlands | -0.005 | (0.025) | -0.073* | (0.038) | -0.054 | (0.047) | 0.001 | (0.039) |
| West Midlands | -0.046* | (0.024) | -0.093** | (0.037) | -0.065 | (0.046) | -0.098** | (0.038) |
| East of England | -0.063** | (0.025) | -0.210*** | (0.038) | -0.144*** | (0.047) | -0.166*** | (0.038) |
| London | 0.027 | (0.025) | 0.016 | (0.040) | 0.109** | (0.048) | 0.054 | (0.040) |
| South East | -0.053** | (0.023) | -0.167*** | (0.036) | -0.106** | (0.045) | -0.115*** | (0.037) |
| South West | -0.065** | (0.025) | -0.215*** | (0.038) | -0.180*** | (0.049) | -0.180*** | (0.040) |
| Residential mobility | -0.009 | (0.014) | -0.016 | (0.024) | -0.016 | (0.029) | -0.009 | (0.023) |
| Birthweight | 0.024*** | (0.009) | 0.043*** | (0.013) | 0.035** | (0.016) | 0.070*** | (0.013) |
| *Birth complications* |  |  |  |  |  |  |  |  |
| Complications, not in special care | -0.012 | (0.010) | -0.005 | (0.015) | 0.001 | (0.018) | -0.015 | (0.016) |
| Complications, in special care | 0.035* | (0.018) | 0.052* | (0.027) | 0.041 | (0.033) | 0.063** | (0.028) |
| Smoking during pregnancy | -0.007 | (0.004) | -0.013** | (0.007) | -0.021*** | (0.008) | -0.016** | (0.007) |
| Alcohol during pregnancy | -0.046*** | (0.014) | -0.108*** | (0.021) | -0.095*** | (0.025) | -0.081*** | (0.021) |
| Parental depression | 0.006*** | (0.002) | 0.009*** | (0.002) | 0.011*** | (0.003) | 0.008*** | (0.002) |
| Child general health | -0.020*** | (0.006) | -0.026*** | (0.010) | -0.027** | (0.011) | -0.016* | (0.010) |
| *Long-standing illness* |  |  |  |  |  |  |  |  |
| Yes, but at most a little bit affected | -0.006 | (0.014) | -0.007 | (0.022) | -0.020 | (0.027) | -0.006 | (0.022) |
| Yes, strongly affected | -0.010 | (0.023) | 0.012 | (0.037) | 0.033 | (0.043) | -0.016 | (0.035) |
| Bracken (Age 3) | 0.007*** | (0.000) | 0.014*** | (0.001) | 0.012*** | (0.001) | 0.015*** | (0.001) |
| BAS vocabulary (Age 3) | 0.001** | (0.001) | 0.007*** | (0.001) | 0.006*** | (0.001) | 0.004*** | (0.001) |
| Externalizing (Age 3) | -0.012*** | (0.001) | -0.029*** | (0.002) | -0.026*** | (0.003) | -0.026*** | (0.002) |
| Internalizing (Age 3) | -0.001 | (0.002) | 0.008** | (0.003) | 0.004 | (0.004) | 0.006* | (0.003) |
| Parents value child independence | -0.001 | (0.005) | -0.010 | (0.007) | -0.001 | (0.008) | -0.011 | (0.007) |
| Parents value child obedience | 0.012** | (0.005) | 0.039*** | (0.007) | 0.036*** | (0.009) | 0.040*** | (0.007) |
| BAS picture (Age 5) - R | 0.002*** | (0.001) | 0.005*** | (0.001) | 0.003*** | (0.001) | 0.008*** | (0.001) |
| BAS vocabulary (Age 5) - R | 0.003*** | (0.001) | 0.007*** | (0.001) | 0.006*** | (0.001) | 0.004*** | (0.001) |
| BAS pattern (Age 5) - R | 0.006*** | (0.001) | 0.016*** | (0.001) | 0.011*** | (0.001) | 0.022*** | (0.001) |
| Externalizing (Age 5) - R | -0.013*** | (0.002) | -0.035*** | (0.003) | -0.037*** | (0.004) | -0.026*** | (0.003) |
| Internalizing (Age 5) - R | -0.003 | (0.002) | 0.005 | (0.004) | 0.006 | (0.004) | 0.001 | (0.004) |
| Educational motivation (Age 5) - R | -0.021*** | (0.007) | -0.047*** | (0.011) | -0.031** | (0.013) | -0.041*** | (0.011) |
| Parents met teacher (Age 5) – R | -0.006 | (0.018) | 0.004 | (0.028) | -0.005 | (0.034) | 0.032 | (0.028) |
| Joint activities (Age 5) – R | 0.003 | (0.006) | 0.008 | (0.009) | 0.021* | (0.011) | 0.000 | (0.009) |
| School Fees (Age 5) – R | -0.114** | (0.056) | -0.212** | (0.093) | -0.176* | (0.097) | -0.164* | (0.093) |
| School change (Age 5) – R | 0.008 | (0.030) | 0.016 | (0.054) | 0.020 | (0.066) | 0.053 | (0.044) |
| BAS reading (Age 7) – R | 0.001** | (0.000) | 0.003*** | (0.001) | 0.005*** | (0.001) | 0.002** | (0.001) |
| BAS pattern (Age 7) – R | 0.003*** | (0.001) | 0.008*** | (0.001) | 0.003*** | (0.001) | 0.013*** | (0.001) |
| NFER math (Age 7) – R | 0.003*** | (0.000) | 0.006*** | (0.001) | 0.003*** | (0.001) | 0.009*** | (0.001) |
| Externalizing (Age 5) – R | -0.010*** | (0.002) | -0.025*** | (0.003) | -0.023*** | (0.004) | -0.020*** | (0.003) |
| Internalizing (Age 5) – R | 0.005** | (0.002) | 0.011*** | (0.003) | 0.004 | (0.004) | 0.012*** | (0.003) |
| Educational motivation (Age 7) – R | 0.016 | (0.013) | 0.036* | (0.021) | 0.011 | (0.024) | 0.044** | (0.020) |
| Parents met teacher (Age 7) – R | 0.005 | (0.022) | 0.009 | (0.037) | 0.031 | (0.039) | 0.029 | (0.036) |
| Reading score in KS 1 – R | 0.010*** | (0.003) | 0.016*** | (0.004) | 0.018*** | (0.005) | 0.009** | (0.004) |
| Writing score in KS 1 – R | 0.009*** | (0.002) | 0.025*** | (0.004) | 0.035*** | (0.005) | 0.008** | (0.004) |
| Math score in KS 1 – R | 0.021*** | (0.002) | 0.055*** | (0.004) | 0.030*** | (0.004) | 0.085*** | (0.004) |
| Parents' educational aspiration (Age 7) – R | 0.021 | (0.036) | 0.105** | (0.050) | 0.104* | (0.060) | 0.021 | (0.049) |
| Joint activities (Age 7) – R | -0.005 | (0.006) | -0.009 | (0.009) | 0.003 | (0.011) | -0.010 | (0.009) |
| School Fees (Age 7) – R | -0.081 | (0.144) | 0.177 | (0.262) | 0.101 | (0.346) | 0.038 | (0.205) |
| School change (Age 7) – R | 0.040*** | (0.015) | -0.009 | (0.024) | -0.015 | (0.028) | 0.028 | (0.025) |
| Top Stream (Age 7) – R | 0.004 | (0.021) | -0.039 | (0.036) | -0.051 | (0.038) | 0.024 | (0.035) |
| Top English set (Age 7) - R | 0.021 | (0.020) | 0.097*** | (0.036) | 0.101*** | (0.039) | 0.049 | (0.034) |
| Top Math set (Age 7) – R | 0.002 | (0.020) | -0.020 | (0.030) | -0.021 | (0.033) | 0.010 | (0.030) |
| Bottom Stream (Age 7) – R | 0.017 | (0.030) | 0.024 | (0.046) | 0.014 | (0.051) | 0.040 | (0.047) |
| Bottom English set (Age 7) – R | -0.024 | (0.028) | -0.010 | (0.042) | -0.007 | (0.046) | -0.018 | (0.044) |
| Bottom Math set (Age 7) – R | -0.005 | (0.028) | -0.026 | (0.037) | 0.001 | (0.045) | -0.020 | (0.036) |
| Verbal similarities (Age 11) – R | 0.002** | (0.001) | 0.007*** | (0.001) | 0.007*** | (0.001) | 0.006*** | (0.001) |
| Externalizing (Age 11) – R | -0.004 | (0.003) | -0.007 | (0.005) | -0.007 | (0.006) | -0.004 | (0.005) |
| Internalizing (Age 11) – R | 0.001 | (0.002) | 0.001 | (0.004) | 0.003 | (0.005) | 0.001 | (0.004) |
| Educational motivation (Age 11) – R | 0.027** | (0.011) | 0.061*** | (0.019) | 0.067*** | (0.022) | 0.020 | (0.018) |
| Parents met teacher (Age 11) – R | -0.003 | (0.025) | 0.016 | (0.038) | 0.002 | (0.044) | 0.007 | (0.038) |
| Reading score in KS 2 – R | 0.022*** | (0.002) | 0.057*** | (0.003) | 0.077*** | (0.004) | 0.016*** | (0.003) |
| Math score in KS 2 – R | 0.019*** | (0.002) | 0.056*** | (0.003) | 0.026*** | (0.004) | 0.092*** | (0.003) |
| Parents' educational aspiration (Age 11) – R | 0.024 | (0.017) | 0.044* | (0.025) | 0.043 | (0.030) | 0.030 | (0.024) |
| No School Fees (Age 11) – R | -0.028 | (0.179) | 0.076 | (0.229) | 0.036 | (0.307) | 0.057 | (0.256) |
| School change (Age 11) – R | 0.003 | (0.013) | 0.016 | (0.018) | 0.011 | (0.022) | 0.020 | (0.019) |
| Top Stream (Age 11) – R | 0.016 | (0.017) | 0.029 | (0.029) | 0.034 | (0.035) | 0.053* | (0.031) |
| Top English set (Age 11) – R | 0.017 | (0.016) | 0.042* | (0.024) | 0.038 | (0.030) | 0.004 | (0.025) |
| Top Math set (Age 11) – R | 0.031* | (0.016) | 0.081*** | (0.024) | 0.052* | (0.029) | 0.095*** | (0.024) |
| Bottom Stream (Age 11) – R | -0.013 | (0.030) | -0.073 | (0.052) | -0.064 | (0.064) | -0.050 | (0.049) |
| Bottom English set (Age 11) – R | -0.017 | (0.023) | -0.011 | (0.035) | -0.048 | (0.043) | -0.003 | (0.032) |
| Bottom Math set (Age 11) – R | -0.028 | (0.022) | 0.009 | (0.030) | 0.035 | (0.035) | -0.034 | (0.031) |
| Vocabulary (Age 14) – R | 0.007*** | (0.002) | 0.030*** | (0.003) | 0.034*** | (0.004) | 0.023*** | (0.004) |
| Externalizing (Age 14) – R | -0.011*** | (0.002) | -0.030*** | (0.003) | -0.028*** | (0.004) | -0.027*** | (0.003) |
| Internalizing (Age 14) – R | 0.000 | (0.002) | 0.006* | (0.003) | 0.004 | (0.004) | 0.009*** | (0.003) |
| Educational motivation (Age 14) – R | 0.027** | (0.012) | 0.126*** | (0.020) | 0.092*** | (0.023) | 0.111*** | (0.019) |
| Parents met teacher (Age 14) – R | -0.019 | (0.018) | 0.003 | (0.026) | 0.005 | (0.032) | 0.002 | (0.024) |
| Parents' educational aspiration (Age 14) – R | 0.002*** | (0.000) | 0.003*** | (0.000) | 0.003*** | (0.001) | 0.003*** | (0.000) |
| No School Fees (Age 14) – R | 0.023 | (0.095) | 0.208 | (0.163) | 0.303 | (0.222) | 0.250 | (0.183) |
| School change (Age 14) - R | -0.005 | (0.023) | -0.083** | (0.035) | -0.085** | (0.043) | -0.035 | (0.037) |
| Constant | 3.471*** | (0.661) | 5.970*** | (0.991) | 7.274*** | (1.184) | 4.090*** | (1.003) |

*Note*. Residualized variables are indicated with an “R” at the end of the variable. N=7,218. Multiple imputed and weighted. *** p<0.01, ** p<0.05, * p<0.1. Reference categories: Absences trajectory: CLA, Ethnicity: White, Parental education: None, Parental class: NSSEC 1, Housing tenure: Owned outright, Region: North East, Birth complications: No, Long-standing illness: No. CLA=Consistently Low Absence, CMAA=Consistently Moderate Authorized Absences, MIUA=Moderately Increasing Unauthorized absences, SIAA=Strongly Increasing Authorized Absences, SIUA=Strongly Increasing Unauthorized Absences.
