# Supplementary material for: Trajectories of school absences across compulsory schooling and their impact on children’s academic achievement: An analysis based on linked longitudinal survey and school administrative data
Source: PLoS One. 2024 Aug 12;19(8):e0306716. doi: 10.1371/journal.pone.0306716 (PMC11318909; doi:10.1371/journal.pone.0306716)
Supplement: Attachment — Submitted filename: Response to Reviewers.docx [file pone.0306716.s011.docx]

# Author response to reviews

We appreciate the editor’s and reviewer’s thorough review of our manuscript and their insightful comments and suggestions. Our opinion is that the resultant revision is significantly clearer and more robust. Following is a discussion of the concerns raised by the editor and one anonymous reviewer, detailing how the manuscript was revised in response to each suggestion.

# Editor

| Comment | Response |
| --- | --- |
| A discussion on policy around absences in England at the time of data collection would be useful - this could be compared with the current policy in the discussion in relation to what you found. | Thank you for the suggestion. We have included a discussion of attendance policy in England during our observation period in the section on absence measurement. There has been no significant change in attendance policy in England in recent years. The most recent development has been a significant increase in absences, particularly following the pandemic, as well as policy responses to reduce absenteeism. In the discussion, we focused our policy implications on post-pandemic student cohorts. |
| A consort diagram in the methods would be useful to understand who was excluded from analysis. For example, were there learners who had missed years of schooling or who did not have GCSEs? | Thank you for your comment. We've included a diagram in the appendix that shows each stage of sample selection. We refer to this diagram in the manuscript. |
| - I limitations section which acknowledges that the data is now approx 8 years out of date (since MCS cohort did GCSEs) would be useful. | Thank you very much for this helpful comment. We have included a limitations section in the discussion, which includes an acknowledgement of the data's historical nature. |

# Reviewer: 1

| Comment | Response |
| --- | --- |
| The introduction sets out the previous literature and the justification for undertaking the current study extremely well. | Many thanks for your comment. We appreciate your positive response about our introduction |
| I am impressed with the careful use of weights, as well as imputation of missing data, in order to get as close a possible to nationally representative estimates for this population. Also the control variables are extremely rich and all very relevant. The use of advanced methods to identify clusters is very commendable. | Thank you. We are delighted that our careful consideration of weighting and imputation are well received. |
| The results are very well presented. I really like the graphs which make it easy to follow. One minor comment is that you may want to put 95% confidence intervals the SD results in the text itself for good measure. | Thank you for your helpful comment. We followed your advice and included 95% confidence intervals in the results description in the text. |
| I am super curious to know whether you found and gender differences in results. Maybe the nice graph in figure two could be done by gender and included as an Appendix and in the text you could refer these as additional analyses and comment on whether girls an boys are similarly affected. | While exploring gender differences in the impact of absence trajectories on achievement would be intriguing, we are limited in our ability to conduct a meaningful analysis in this regard due to sample sizes. Some trajectories have relatively small sample sizes, making it statistically challenging to differentiate results by gender effectively because of low statistical power. We believe that an analysis of gender differences will be possible using the full National Pupil Database rather than the sub-sample (linked with MSC) chosen for our study. We have included this as an area for future research in our discussion. |
| If space permits, if would be good to highlight the huge increase in absence from before the pandemic: <https://www.gov.uk/government/statistics/pupil-absence-in-schools-in-england-2018-to-2019> compared to after the pandemic: <https://explore-education-statistics.service.gov.uk/find-statistics/pupil-absence-in-schools-in-england> This would highlight and support the importance of your findings for the current generation of school children who miss school. | Many thanks for this helpful comment. We have added a paragraph on rising school absences after the pandemic and our findings’ implications for post-pandemic student cohorts in the discussion. |
| Although the paper and the study is methodologically very sound, it would be good to see you list some study limitations. E.g. that although significant efforts have been made to control for confounding factors, because this is an observational study, care has to be taken with interpreting results a causal. | Thanks very much for this comment. We agree that the results must be interpreted with caution when it comes to causal claims. We have added a section on study limitations in the discussion, including caution with causal inference. |
| I know that the data cannot be published due UK Data Service licence restrictions, and you have stated this. However, I suggest that you make your analytical code available to readers on GitHub for example. The research community could really benefit from this when conducting a similar study. | Thank you for your comment. Our code, like the data, is currently only accessible in a Secure Access environment through the UK Data Service. We are in discussions with the data provider about whether the syntax files can be exported in the future. If possible, we will make our code available through the Open Science Framework, as we have done with previous research based on publicly available secondary data. |
